# Synergistic effects of electroactive antibacterial material and electrical stimulation in enhancing skin tissue regeneration: A next‐generation dermal wound dressing

**DOI:** 10.1111/srt.13465

**Published:** 2023-10-26

**Authors:** Xi‐Liang Zang, Fei Gao, Zhao Zhang, Lin‐Hua Shen, Yue‐Hai Pan

**Affiliations:** ^1^ Qingdao University Qingdao Shandong China; ^2^ Department of Trauma Microsurgery 970 Hospital of the PLA Joint Logistic Support Force Yantai Shandong China

**Keywords:** antibacterial polypeptide, biocompatible polymer, chitosan, reduced graphene oxide, skin defect

## Abstract

**Objective:**

We aimed to develop an electroactive antibacterial material for the treatment of skin wound diseases.

**Method:**

To this aim, we modified chitosan (CS), a biocompatible polymer, by coupling it with graphene (rGO) and an antimicrobial polypeptide DOPA‐PonG1. The material's effect on skin injury healing was studied in combination with external electrical stimulation (EEM). The structure, surface composition, and hydrophilicity of the modified CS materials were evaluated using scanning electron microscopy (SEM), Fourier‐transform infrared spectroscopy (FTIR), and contact angle measurements. We studied NIH3T3 cells cultured with modified materials and subjected to EEM to assess viability, adhesion, and tissue repair‐related gene expression.

**Results:**

SEM data demonstrated that rGO was distributed uniformly on the surface of the CS material, increasing surface roughness, and antimicrobial peptides had minimal impact on surface morphology. FTIR confirmed the uniform distribution of rGO and antibacterial peptides on the material surface. Both rGO and DOPA‐PonG1 enhanced the hydrophilicity of CS materials, with rGO also improving tensile strength. The dual modification of CS with rGO and DOPA‐PonG1 synergistically increased antibacterial efficacy. Cellular events and gene expression relevant to tissue repair process were enhanced by these modifications. Furthermore, EEM accelerated epidermal regeneration more than the material alone. In a rat skin wound model, DOPA‐PonG1@CS/rGO dressing combined with electrical stimulation exhibited accelerated healing of skin defect.

**Conclusion:**

Overall, our results demonstrate that CS materials modified with rGO and DOPA‐PonG1 have increased hydrophilicity, antibacterial characteristics, and tissue regeneration capacities. This modified material in conjunction with EEM hold promise for the clinical management for dermal wounds.

## 1 INTRODUCTION

The skin possesses inherent self‐healing capabilities.^1^ However, in some conditions such as large wounds or diabetic ulcers, these abilities may turn out to be inadequate, necessitating the use of wound dressings to promote healing. Traditional dressings, such as gauze, have limitations such as poor moisture retention and wound surface adhesion. To overcome these deficiencies, researchers have turned to natural and synthetic materials for developing improved wound dressings.^2^

Chitosan (CS) and its derivatives possess several desirable properties for wound dressing including biocompatibility, biodegradability, broad‐spectrum antibacterial activity, hemostatic properties, and anti‐adhesive characteristics.^3^ However, pure CS materials have low mechanical strength that hinders the shape and structural adaptability of the materials necessary during the healing process. Moreover, CS has limited intrinsic antibacterial ability that restricts its efficacy in stimulating skin wound regeneration.^4^ Consequently, researchers are actively exploring functional modifications of CS materials to address these limitations.

Reduced graphene oxide (rGO), which is produced from graphene is the ideal functional modifier for materials due to its excellent mechanical rigidity, biocompatibility, and a unique two‐dimensional structure. Electrical activity of CS and other materials is greatly increased by the addition of rGO, and promote collagen deposition in wound tissue, thereby accelerating wound healing.^5^ ^6^ Additionally, electroactive materials possess effective charge conduction.^7^ When combined with electrical stimulation therapy, they offer precise regulation of cell behaviors such as proliferation, adhesion, and differentiation required for wound healing.^8^

An important factor affecting the therapeutic efficacy of a drug for skin wound is bacterial infection that leads to increased exudate at the wound site, inhibit the formation of granulation tissue, and thus inhibit wound healing.^9^ To prevent bacterial infection, antibiotics such as penicillin and methicillin are commonly used. However, due to the abuse of antibiotics and the emergence of drug‐resistant bacteria, it is necessary to find an alternative to broad‐spectrum antibiotics. In recent years, antibacterial peptides including defensins, lysozyme, and lactoferrin have shown promise in the treatment of wound infections.^10^, ^11^ These antibacterial peptides are ubiquitous in all eukaryotic organisms and are the basic elements of the immune system. These peptides kill bacteria by inducing the hydrolysis of bacterial walls, destroying the integrity of bacterial membranes and changing the lipid distribution of phospholipid membranes. The major advantage of antimicrobial peptides is their ability to kill bacteria without leading to the development of drug resistance, which is a significant concern with traditional antibiotics. Moreover, antibacterial peptides can be engineered and modified by introducing specific gene sequences, enabling precise regulation of their physical and chemical properties. This customization allows researchers to optimize the antimicrobial activity, stability, and other characteristics of these peptides, to increase expanding their potential applications.^11^

In this regard, Zhan Jing et al. developed biodegradable polymers in combination with bioactive components for the treatment of skin wounds.^12^ The adhesion mechanism observed in marine mussels inspired this method. The adhesive protein of the marine mussle, 3,4‐Dihydroxyphenylalanine (DOPA) was incorporated into basic fibroblast growth factor (bFGF) and ponericin G1 (PonG1) by tyrosine hydroxylation.

Zhan Jing et al. then combined DOPA with an antimicrobial peptide ponericin G1 (PonG1) by incorporating a DOPA sequence into the PonG1 peptide. DOPA‐PonG1 exhibits prolonged stability when attached to materials, and effectively inhibits the growth of pathogenic microorganisms by targeting bacterial cell walls and disrupting bacterial membranes.^12^ In this study, we combined electroactive antibacterial materials with electrical stimulation, and investigated its effect on the cutaneous injuries in rat models. We further evaluate the combined effects of these two approaches on cellular behavior and gene expression involved in tissue regeneration. By synergistically harnessing the benefits of active antibacterial properties and electrical stimulation, we aimed to provide novel therapeutic strategies for the management of cutaneous lesions that are difficult to heal.

## 2 MATERIALS AND METHODS

### 2.1 Reagents

We procured CS (degree of deacetylation = 98%) (Aladdin Reagent Company, China), rGO (China Graphene Research Institute, China), thiazolium blue (Sanghai LigatechBiotechnology Co., Ltd., China), PonG1 (Beijing Liuhe Huada Gene Technology Co., Ltd., China), fluorescein isothiocyanate (FITC, Sigma‐Aldrich, USA), tyrosinase (Sigma‐Aldrich), vitamin C (Beijing Suolaibao Biotechnology Co., Ltd., China). Cell culture media (DMEM), fetal bovine serum (FBS), and trypsin were procured from Life Technologies Corp, USA.

### 2.2 Preparation of DOPA‐PonG1@CS/rGO electroactive antibacterial material

Two gram CS and 40 mg rGO were dissolved in 100 mL (0.5% v/v) acetic acid solution, and then spread the mixture evenly on a glass plate. After drying for 48 h, the CS/rGO composite was thoroughly washed with alkaline deionized water. Finally, the solidified CS/rGO material was freeze‐dried and placed in a vacuum oven for further use.

#### 2.2.1 Synthesis of DOPA‐PonG1

We introduced a tyrosine linker (YKYKY) to the N‐terminus of PonG1 (N'‐WKDWAKKA‐GGWLKKKGPGMAKAALKAAMQ‐C') to synthesize YKYKY‐PonG1. The final product was synthesized by the Beijing Liuhe Huada Gene Technology Ltd, China.

#### 2.2.2 Surface loading of DOPA‐PonG1 materials

The CS/rGO and CS flakes with a diameter of 1 cm were immersed in the DOPA‐PonG1 solution for 24 h, and then, the materials of each group were washed with PBS solution and freeze‐dried for later use.

#### 2.2.3 Material characterization

Physical observations of shape and color of materials such as CS, CS/rGO, DOPA‐PonG1@CS, and DOPA‐PonG1@CS/rGO were noted and photographs taken.

Scanning electron microscopy (SEM, XL30ESEM‐FEG, Japan) was used to examine the surface morphology of the produced materials in each group before and after gold spraying. Fourier transform infrared spectroscopy (FTIR, IFS66V/S vacuum type, Bruker, Germany) was used to detect the surface chemical composition across a scanning range of 500–4000 cm^−1^, and an x‐ray diffractometer (XRD, Bruker D8 Advance, Germany) was used to determine the surface lattice structure of the samples, collecting diffraction data from 5° to 80°. The hydrophilicity of the materials was determined by adding 2 μL of ultrapure water onto the surface of each group and measuring the contact angle using a contact angle measuring instrument (VCA 2000, Germany).

*Staphylococcus aureus* and *Escherichia coli* were used as experimental strains to examine the antibacterial efficacy of each material. After co‐culturing a bacterial suspension at a concentration of 5 × 10^5^ CFU/mL with each material group for 12 h, the absorbance value of the bacterial solution at 600 nm was measured after the removal of the liquid.

Thin slices of 20 × 10 cm were made for each material group to measure mechanical performance. Tensile testing was performed on the samples using a multifunctional mechanical tester, and the highest tensile strength value was recorded.

### 2.3 Biological assays

NIH3T3 cells (The Shanghai Cell Bank of CAS, China) were incubated with DMEM medium (supplemented with 10% FBS). Cells were seeded at the confluence of 2×10^4^ cells/well in 24‐well plates and incubated at 37°C with 5% CO_2_, and 95% humidity.

The electrical stimulation device consists of a digital function signal generator (Hantai HDG2002B, China), a digital oscilloscope and a 24‐well plate modified with platinum electrodes. To generate a uniform electric field in the culture medium, a pair of L‐shaped platinum electrodes, 10 mm apart, were placed on the lid of a 24‐well cell culture plate. The digital function signal generator uses platinum electrodes to stimulate cells with different electrical frequencies. Electrical stimulation parameters were adjusted by a digital oscilloscope. The cells were cultured under different electrical voltage stimulation, and viability was assessed by the MTT (3‐(4,5‐dimethylthiazol‐2‐yl)−2,5‐diphenyltetrazolium bromide) method.

To assess the effect of electroactive antibacterial materials combined with electrical stimulation on cell viability and adhesion, cells were seeded (3×10^4^ cells/well in a 24‐well plate). Electrical stimulation was applied to the cells, and the viability was measured by MTT method at 1, 4, and 7 days.

To study adhesion, cells were first grown on the material surfaces for 3 days, and following the electrical stimulation, the cells were fixed with 4% paraformaldehyde and stained with FITC to observe the cytoskeleton and DAPI to visualize the nucleus by fluorescence microscopy (Zeiss Company, Germany).

To assess the effect of electroactive antibacterial materials in combination with electrical stimulation on the expression of tissue repair‐related genes, cells were cultured on the surfaces of each material group for 7 days. The cells were then lysed with Trizol and total RNA was extracted. From the total RNA, cDNA library was synthesized (Kit name and manufacturer details) followed by PCR (Kit name and manufacturer details) using gene‐specific primers given in Table 1.

**TABLE 1:** PCR primers.

Gene name	Upstream primer	Downstream primer
COL‐1	5‐CTGAAATGTCCCACCAGCC‐3	5‐GTCCGATGTTTCCAGTCTGC‐3
VEGF	5‐CCTTCAGCTCGCTCCTCC‐3	5‐GAAGATGAGGAAGGGTAAGCC‐3

### 2.4 Animal experiments

Fifteen male SD rats (220–260 g) were purchased from Liaoning Changsheng Biological Company (STATE AND COUNTRY) and used for investigating full‐thickness skin defects. Rats were divided into five equal groups that received CS, DOPA‐PonG1@CS, CS/rGO, DOPA‐PonG1@CS/rGO, and DOPA‐PonG1@CS/rGO+ electrical stimulation (ES). All animal experiments were approved by the ethics committee of Qingdao University

Rats were anesthetized with chloral hydrate, and the dorsal areas were shaved and disinfected. Circular wound with a diameter of 1 cm in the skin was made by removing the epidermis, dermis, and subcutaneous connective tissue. Subsequently, the wounds were treated with various materials and ES, with the replacement of materials every 3 days. The wounds were visually examined and photographed at 0, 3, 6, and 9 days post‐operation, and the area was measured to calculate the wound healing rate. After 9 days, epidermal tissue from each experimental group was collected, fixed in paraformaldehyde, embedded in paraffin, and sectioned. HE and Masson staining were performed on the tissue sections to study tissue regeneration using a microscope.

### 2.5 Statistical analysis

The statistical software Origin 8.0 was used for data processing. One‐way ANOVA (one‐way ANOVA) was used for comparison between groups, and *p* < 0.05 indicated a statistically significant difference.

## 3 RESULTS

### 3.1 General observations

**FIGURE 1:**
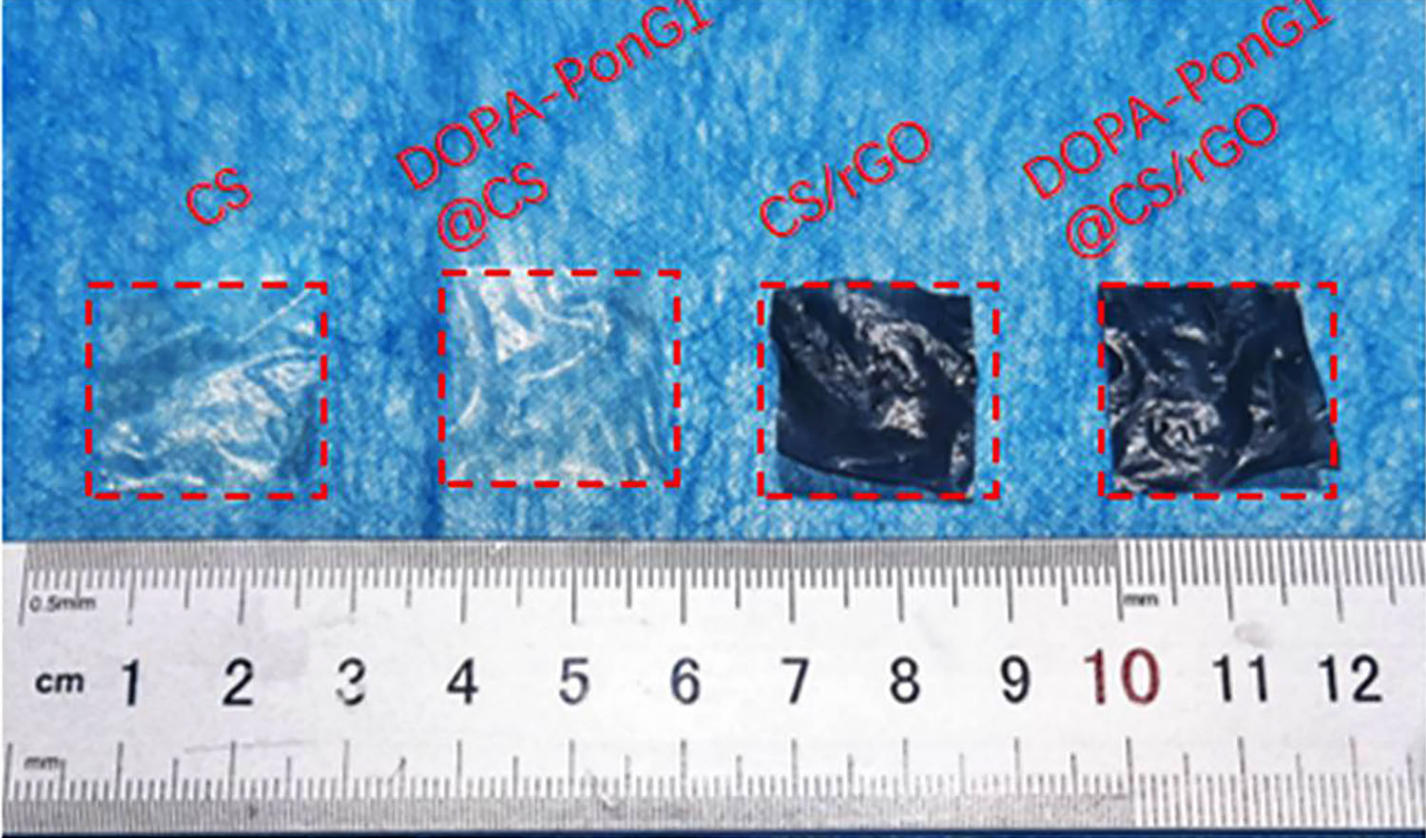
General morphology of CS, DOPA‐PonG1@CS, CS/rGO, and DOPA‐PonG1@CS/rGO.

Pure CS material appeared as transparent flakes. When rGO was added, the surface color of the material turned black, indicating an opaque state. Furthermore, when DOPA‐PonG1 was added to the CS material, the surface properties of the materials in each group did not undergo significant changes, thus implying that the addition of DOPA‐PonG1 did not have a pronounced effect on the surface characteristics, such as color or visual appearance, of the materials (Figure 1).

### 3.2 SEM studies

**FIGURE 2:**
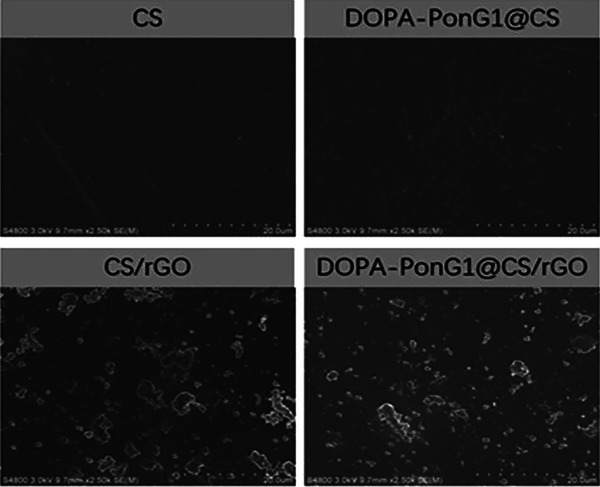
Surface microstructure of CS, DOPA‐PonG1@CS, CS/rGO, and DOPA‐PonG1@CS/rGO. Bar −20 μm.

The pure CS group displayed a smooth and flat surface when observed under the SEM. However, after the addition of rGO nanoparticles to the CS material, its surface became rough, and numerous agglomerated particles were observed. However, the microstructure of the material surface did not change after loading DOPA‐PonG1 (Figure 2). These data suggested that the presence of DOPA‐PonG1 had minimal impact on the surface characteristics.

### 3.3 FTIR studies

**FIGURE 3:**
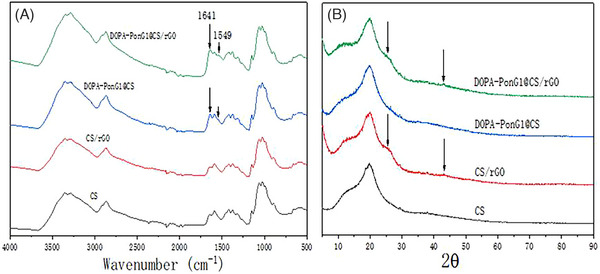
Chemical characterization of various materials: (A) FTIR and (B) XRD analyses.

CS material had characteristic peaks at 897  and 1154 cm^−1^, which are associated with the chitosan structure.^12^ A peak at 1561 cm^−1^, corresponding to the δ(N‐H) vibration, was also observed. Upon the addition of rGO to the CS material, there was no significant change in the material's FTIR spectral profile. However, after loading DOPA‐PonG1 onto the CS material's surface, the peak intensities at 1641  and 1549 cm^−1^, corresponding to the stretching vibration of C = O, bending of N‐H, and stretching vibration of C‐N, were significantly increased. These peaks are characteristic of polypeptides, suggesting that DOPA‐PonG1 was successfully incorporated onto the CS material (Figure 3A).

### 3.4 XRD studies

Upon addition of rGO to the CS material, a peak appeared at a 2θ position of approximately 25°. This specific peak is a characteristic feature of rGO, indicating the successful incorporation and presence of rGO within the CS matrix. By contrast, when DOPA‐PonG1 was added to the CS material, there was no significant change in the XRD pattern, suggesting that the addition of DOPA‐PonG1 had no effect on the crystalline structure or overall composition of the CS material (Figure 3B).

### 3.5 Hydrophilicity studies

**FIGURE 4:**
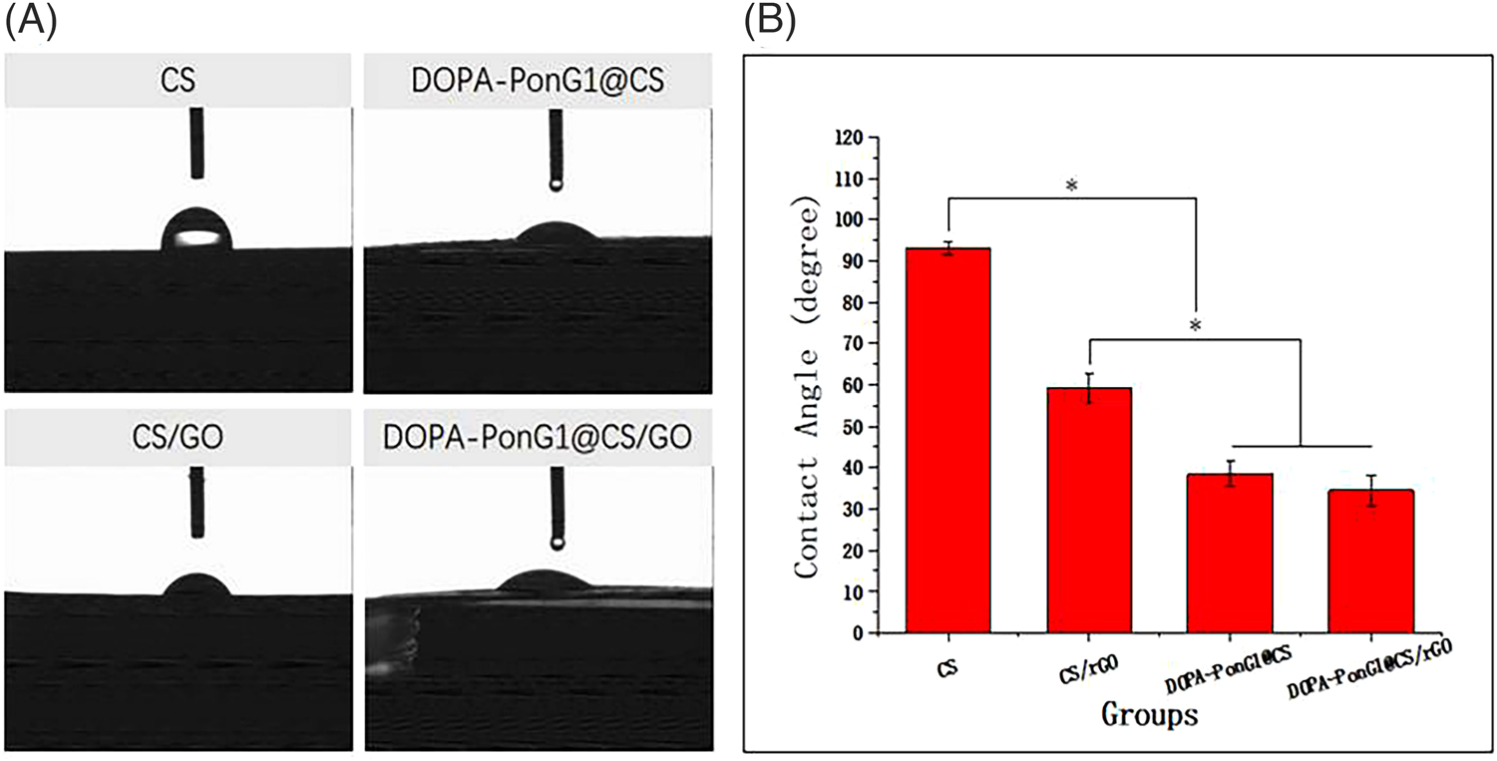
Surface property analysis of various materials by contact angle analysis. (A) Representative photograph of surface of each material and (B) quantification of the data for all groups; *n* = 3; ^*^ *p* < 0.05.

We conducted the contact angle test to assess the hydrophilicity of the CS material before and after modifications. The initial contact angle of the CS surface was found to be 92.9 ± 1.6°, indicating a relatively hydrophobic nature of the material. After the addition of rGO to the CS material, the contact angle decreased to 59 ± 3.51°, suggesting an improvement in hydrophilicity compared to the pure CS material. Similarly, when DOPA‐PonG1 was added to the CS material, the contact angle further reduced to 38.6 ± 2.96°, indicating a significant improvement in hydrophilicity compared to both pure CS and CS‐rGO composites. Furthermore, when both rGO and DOPA‐PonG1 were used to modify the CS material simultaneously, the contact angle further decreased to 34.47 ± 3.58°, indicating excellent water affinity and superior hydrophilicity (Figure 4).

### 3.6 Study of the mechanical properties

**FIGURE 5:**
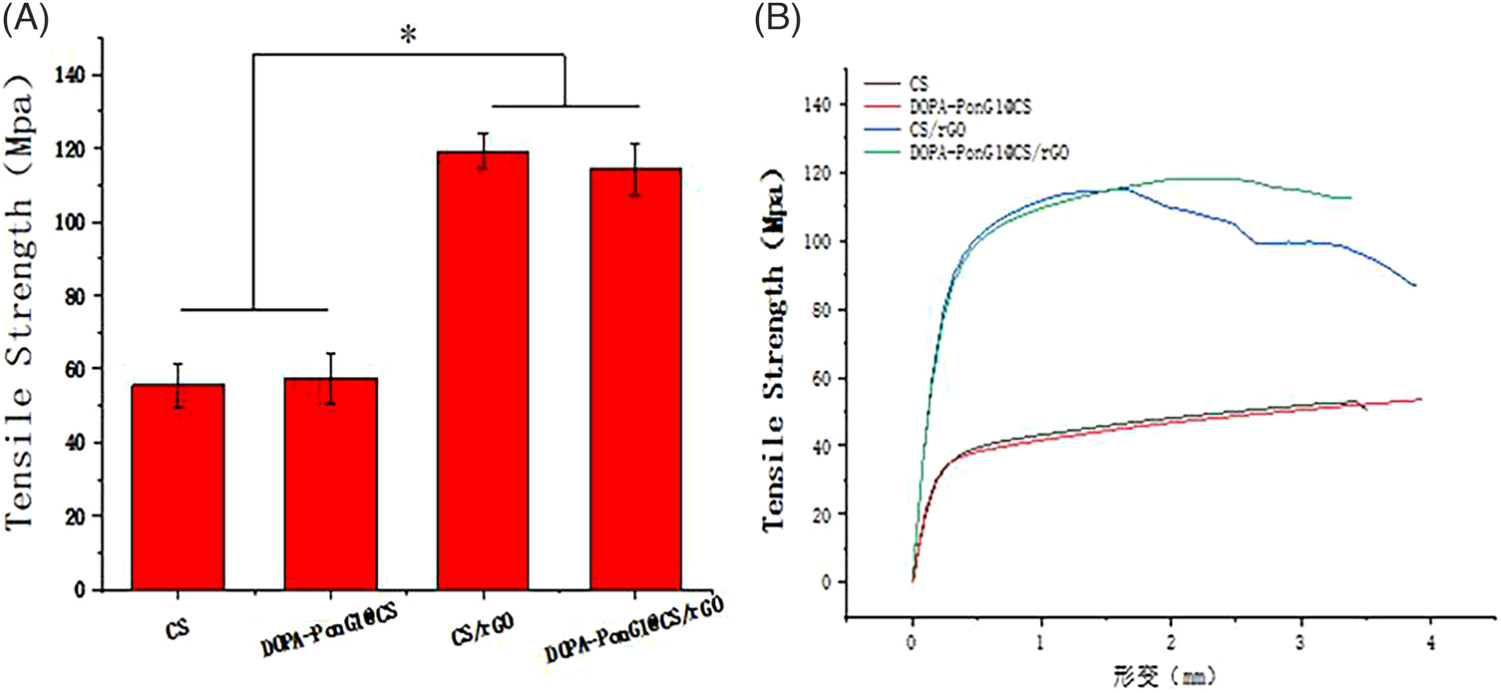
Mechanical property analysis of various materials. (A) Tensile curve map of each material and (B) quantification of the data for all groups; *n* = 3; ^*^ *p* < 0.05.

We evaluated the impact of surface loading with DOPA‐PonG1 and the incorporation of rGO on its mechanical performance. We found that surface loading of DOPA‐PonG1 did not have a significant effect on the mechanical properties of the material. However, when rGO was added to the material, the tensile strength of the material increased from 55.4 ± 6.13  to 119.2 ± 4.62 MPa, indicating a significant enhancement in mechanical properties (Figure 5).

### 3.7 Assessment of anti‐bacterial effect

**FIGURE 6:**
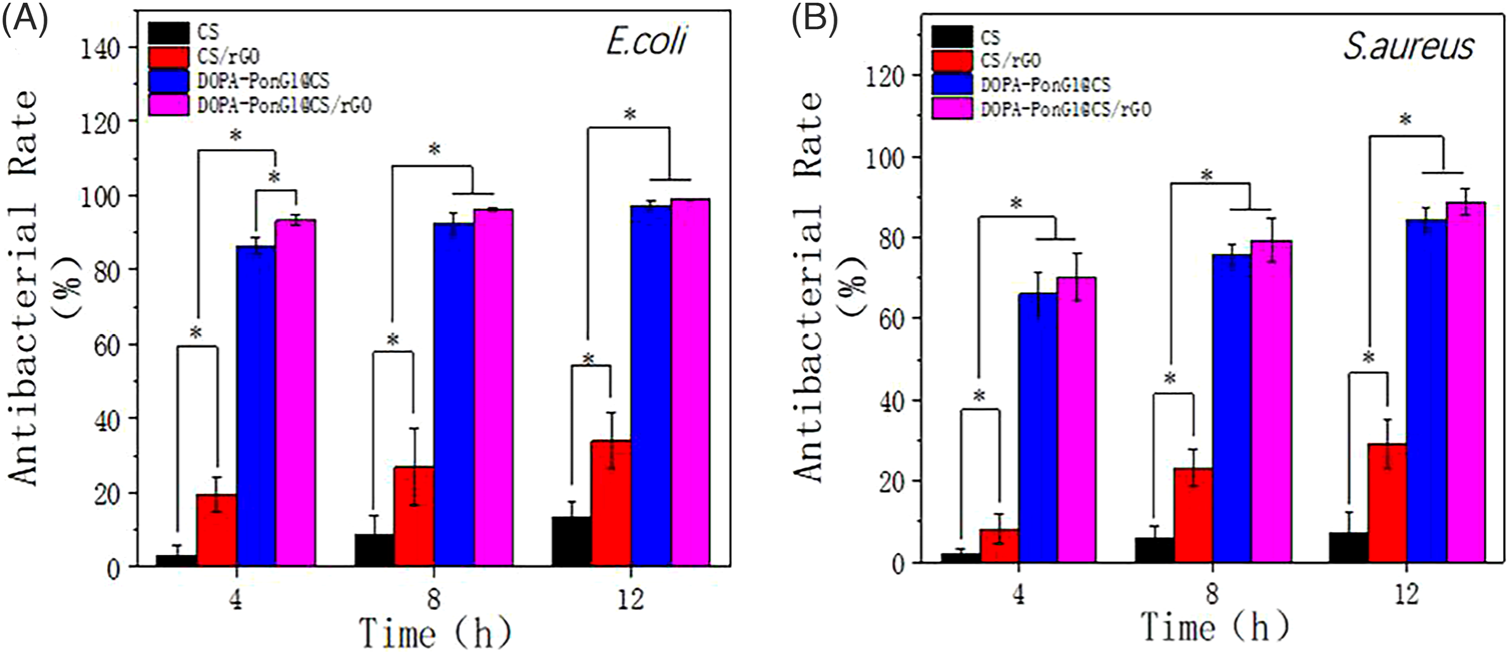
Antibacterial properties of the materials were evaluated by measuring the optical density (OD) of *Escherichia coli* (A) and *Staphylococcus aureus* (B) cultures co‐incubated with different materials for 4, 8, and 12 h; *n* = 3; ^*^ *p* < 0.05.

The antibacterial effect of the materials was evaluated by testing their ability to inhibit the growth of pathogenic bacteria. The antibacterial rate in the CS group was approximately 10% after 12 h of co‐culture with the pathogenic bacteria (*Escherichia coli* and *Staphylococcus aureus*). The addition of rGO to the CS material increased the antibacterial action by approximately 30%. The addition of DOPA‐PonG1 to the material increased the antibacterial efficacy to 90% (*p* < 0.05) when compared to that found in the CS and CS/rGO groups (Figure 6).

### 3.8 Effects on cultured cells

**FIGURE 7:**
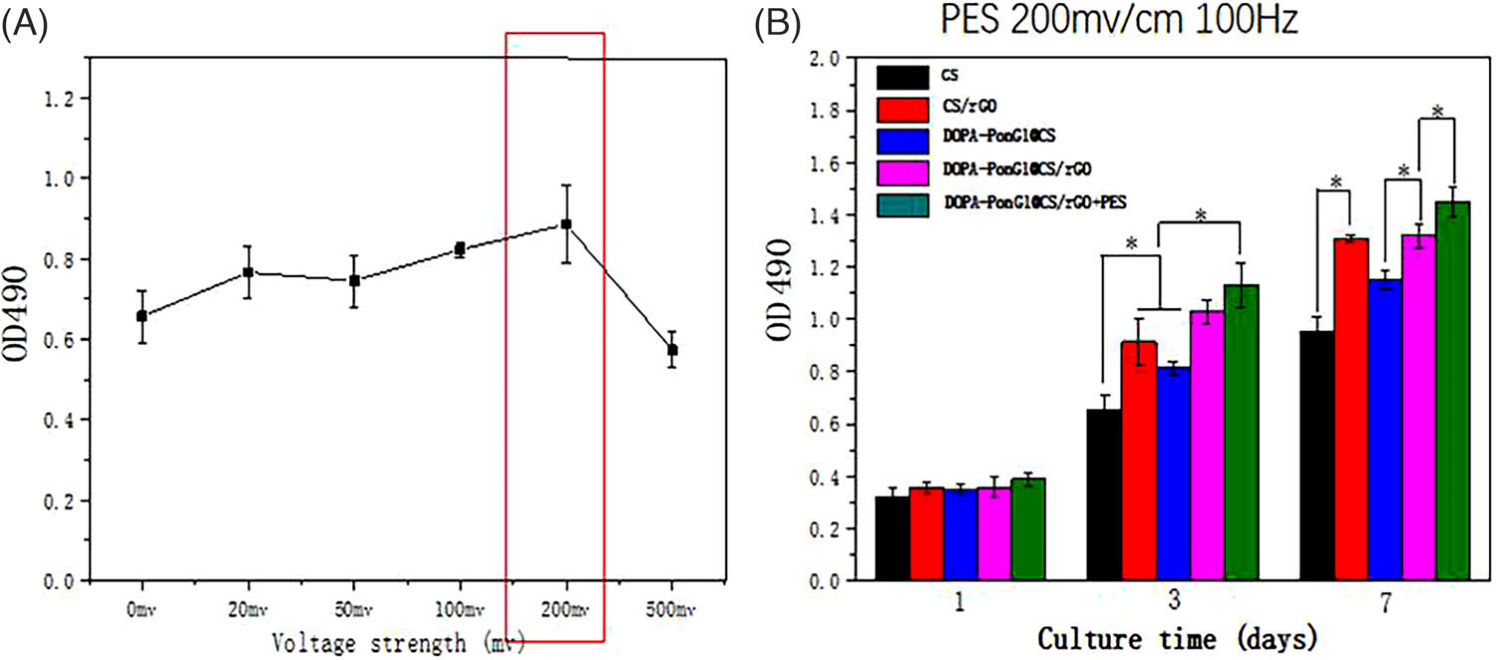
Analysis of cell viability. (A) Cell viability rate of NIH3T3 cells under various electrical stimulation parameters; *n* = 3; ^*^ *p* < 0.05. (B) Viability rate of NIH3T3 cells at 1, 3, and 7 days when treated with different materials under optimal electrical stimulation conditions; *n* = 3; *p* < 0.05.

We next applied ES to NIH3T3 cells with a range of voltages to determine the best frequency for promoting cell viability assessed by MTT assay. After 3 days of culture, the group exposed to 200 mV displayed the highest OD value, indicating the most significant viable number of cells among the different voltage groups. According to the MTT results, we selected the 200mv electrical stimulation voltage to treat NIH3T3 cells in the subsequent experiments (Figure 7A).

Next, NIH3T3 cells were grown on various surfaces while being stimulated electrically. On day 3, all groups except the CS group displayed a marked increase in cell viability. On day 7, the CS/rGO group demonstrated greater viability, more than the DOPA‐PonG1@CS group. Remarkably, the DOPA‐PonG1@CS/rGO+ES group showed the highest viability compared with the other two groups (*p* < 0.05) (Figure 7B). These results show that electrical stimulation, DOPA‐PonG1, CS, and rGO promote cell viability, indicating its potential for tissue repair and regenerative applications.

**FIGURE 8:**
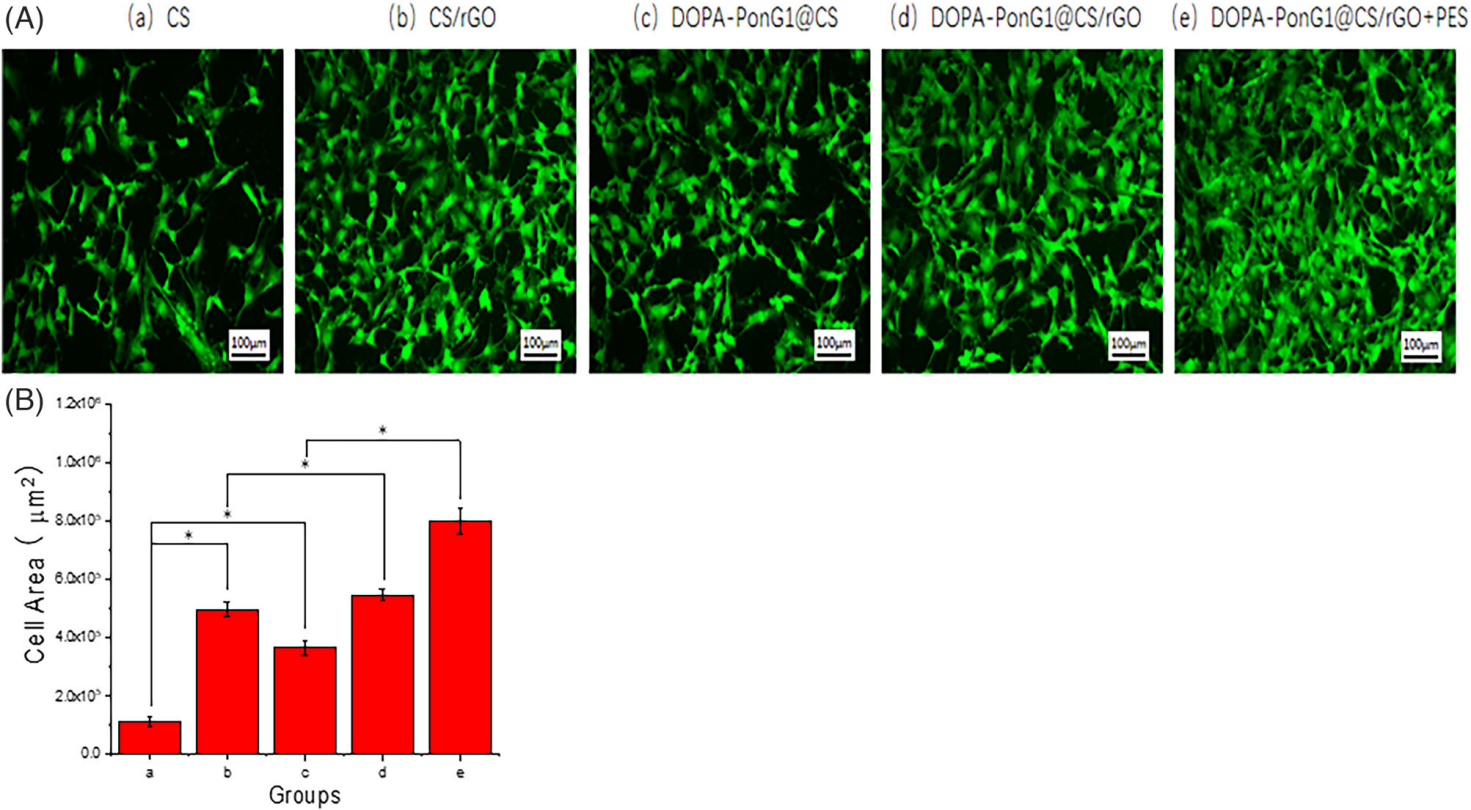
Analysis of cell adhesion. Adhesion of NIH3T3 cells on different surfaces and under optimal electrical stimulation conditions, bar – 100 μM. (B) Quantification of the aggregate cellular area within the field of view; ^*^ *p* < 0.05.

We next studied cell adhesion and growth using FITC staining of the cytoskeletal structure of NIH3T3 cells. Substantial number of adherent cells were seen in all groups after 3 days of different conditions. Cell adhesion increased in all experimental groups after the administration of rGO. Notably, compared to the other groups, the NIH3T3 cells in the DOPA‐PonG1@CS/rGO+ES group showed a tighter connection and a larger area covered by the adherent cytoskeleton. These results suggested that the combination of DOPA‐PonG1, CS, rGO, and electrical stimulation promoted cell adhesion and cytoskeletal organization, which are essential for tissue repair and regeneration (Figure 8A). Upon quantification of the aggregate cellular area within the field of view, we observed that cell adhesion increased in all groups compared with CS. Notably, the the DOPA‐PonG1@CS/rGO+ES group exhibited the most extensive total cell area when compared to the other groups (Figure 8B).

**FIGURE 9:**
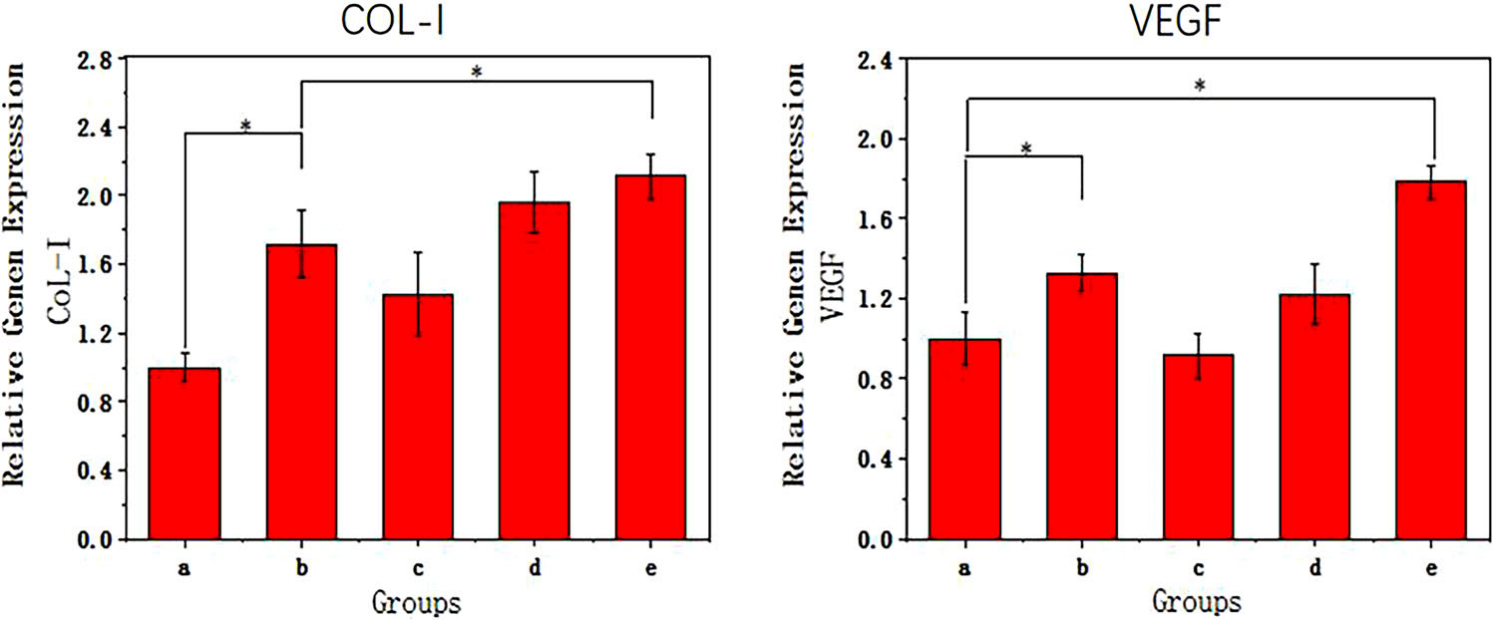
Determination of gene expression related to tissue repair. The qPCR analyses of Col‐1 and VEGF in NIH3T3 cells in response to individual material; (A) CS,(B) CS/rGO, (C) DOPA‐PonG1@CS, (D) DOPA‐PonG1@CS/rGOF, and (E) DOPA‐PonG1@CS/rGO+ES. *N* = 3, ^*^ *p* < 0.05.

We performed qPCR to assess the expression of Col‐1, the osteogenic differentiation‐related gene and VEGF, the angiogenic gene. The results showed that both rGO and DOPA‐PonG1 modified materials significantly increased the expression of COL‐I gene compared to the CS group. rGO had a more pronounced effect in upregulating COL‐I gene. VEGF expression was upregulated more in the rGO group compared to the CS group, whereas the DOPA‐PonG1 modification alone did not have any enhancing effect on the expression of this gene. Among all the experimental groups, the DOPA‐PonG1@CS/rGO+ES group showed the highest upregulation of COL‐I and VEGF (Figure 9). These findings suggested that the combined modification of DOPA‐PonG1, CS, and rGO, along with electrical stimulation are effective in upregulating the genes that promote osteogenic differentiation and angiogenesis.

### 3.9 Wound healing studies in animals

**FIGURE 10:**
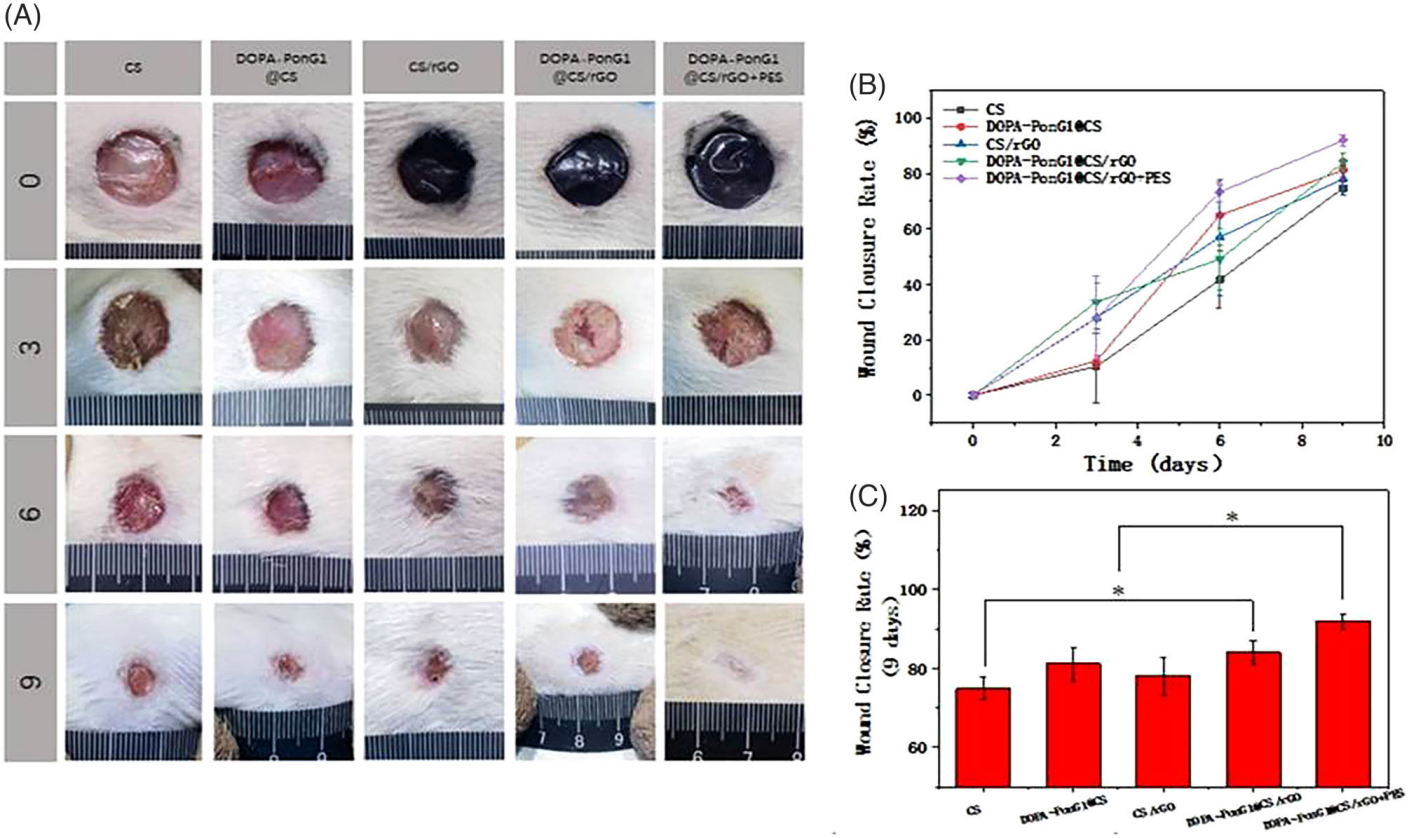
The analysis of skin wound repair in rats. (A) The visual appearance of wounds. (B) The epidermal healing rate of rats at different time points. (C) The skin healing rate of rats in each experimental group after 9 days. *N* = 3, ^*^ *p* < 0.05.

We assessed the wound healing potential of different materials combined with electrical stimulation in rats with full‐thickness skin damage model obtained by making circular wound of ∼1 cm in diameter on the skin surface. Beginning on day 3 post‐surgery, the wound areas in each experimental group steadily decreased, with the CS group consistently showing bigger wound areas than the other groups. On day 9 post‐surgery, the DOPA‐PonG1@CS/rGO+ES group had the smallest wound area of all experimental groups, closely resembling normal skin tissue and indicating the highest efficient regenerative outcome (Figure 10A).

We next studied the wound healing rates at various time intervals. On the third post‐surgery day, the wound surfaces in the CS/rGO, DOPA‐bFGF@CS/rGO, and DOPA‐PonG1@CS/rGO+ES groups were considerably lower than in the CS and DOPA‐PonG1@CS groups. These groups maintained a favourable repair effect throughout time, while the DOPA‐PonG1@CS group also demonstrated significantly enhanced epidermal healing, with a healing rate of 66.8%, which was significantly greater than the CS group. The DOPA‐PonG1@CS/rGO+ES group had the highest wound healing rate on day 9, reaching 91.9%, which was significantly higher than the other groups (Figure 10B, C).

We studied the newly generated tissue from wound healing site by HE and Mason staining with the aim to gain insights into the effectiveness and quality of the wound healing process in the experimental groups. The CS group showed the thickest epidermis, followed by relatively smaller epidermal thickness in the CS/rGO and DOPA‐PonG1 groups. DOPA‐PonG1@CS/rGO and DOPA‐PonG1@CS/rGO+ES groups had skin thickness that was similar to normal skin thickness. The DOPA‐PonG1@CS/rGO and DOPA‐PonG1@CS/rGO+ES groups had denser subcutaneous tissues, more new capillaries, and better extracellular matrix integrity than the other groups.

**FIGURE 11:**
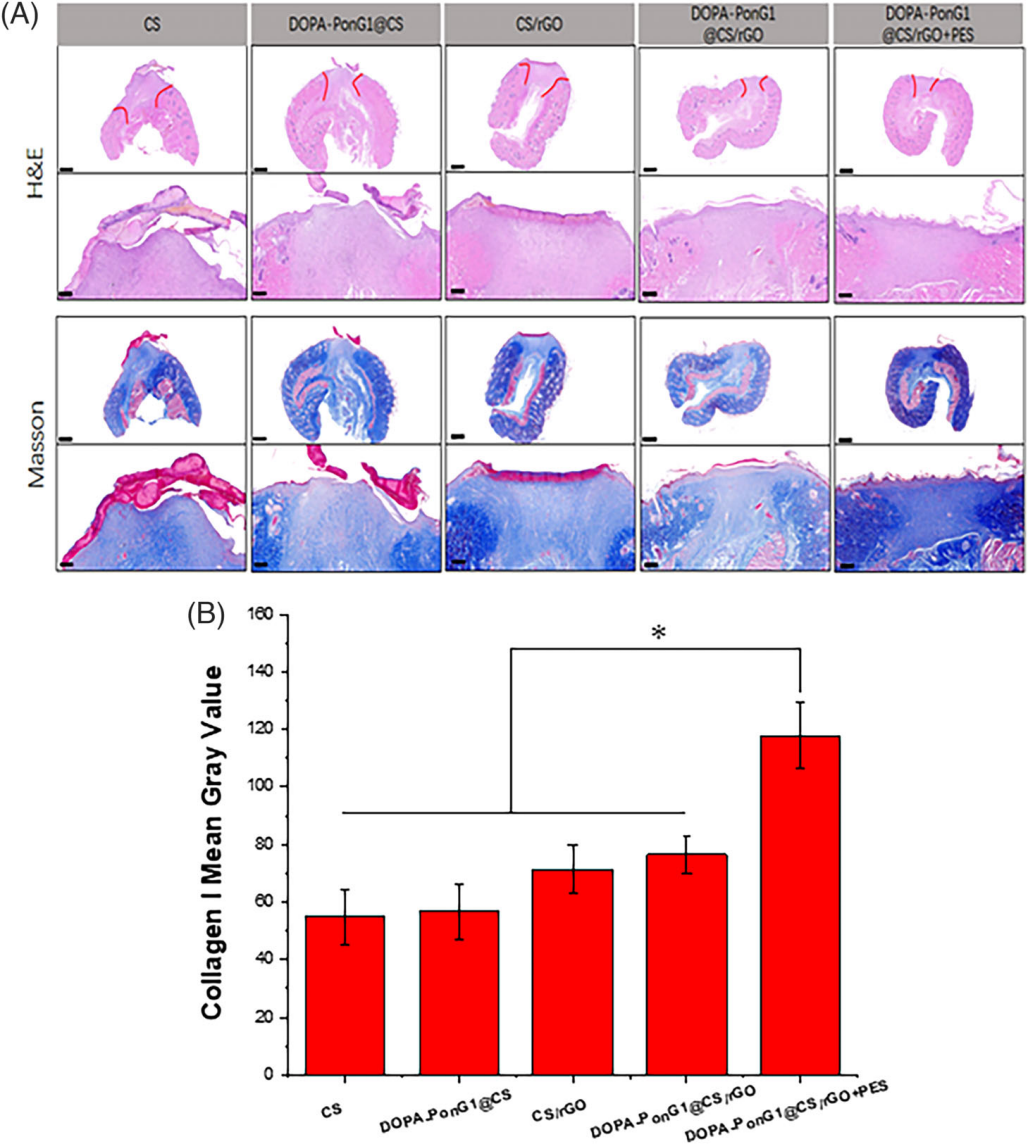
Histomorphological evaluation of wound regeneration for different experimental groups on the 9 day through H&E and Masson's trichrome staining. (B) Collagen I at the wound repair site was quantified using ImgaeJ software; ^*^ *p* < 0.05 compared with other groups.

Masson staining indicated varied levels of collagen fibers in the newly generated tissues of each group. The collagen level was decreased, and fiber arrangement was disorganized in the pure CS group. The PonG1@CS group had collagen content comparable to CS. The CS/rGO and DOPA‐PonG1@CS/rGO groups had higher collagen deposition with more organized arrangement. The DOPA‐PonG1@CS/rGO+ES group had the most collagen deposition, which was characterized by dense and regular orientation and distribution (Figure 11A). The average gray value in the DOPA‐PonG1@CS/rGO+ES group was higher than the other groups (Figure 11B). Overall, these data demonstrated that the DOPA‐PonG1@CS/rGO+ES group had better quality of new skin tissue, implying improved tissue regeneration and extracellular matrix remodeling.

## 4 DISCUSSION

There is growing interest in using natural or synthetic materials for wound dressing preparation in order to overcome these restrictions.^13^ Because of its remarkable biodegradability and compatibility with living cells, chitosan is a well‐known material for wound repair that is achieved by promoting fibroblast migration and collagen deposition.^14^ Moreover, because chitosan is inexpensive and easily modifiable material, it is suitable for producing skin wound dressings. However, it has some limitations, including inadequate absorption, sensitivity to degradation, poor mechanical qualities, and limited antibacterial action.^15^ To address these constraints, researchers have investigated the integration of functionally active components into chitosan materials to improve their tissue regeneration capacities.

In this study, we examined the effects of adding rGO and DOPA‐PonG1 on the physical and chemical properties of the materials. The uniform dispersion of rGO and DOPA‐PonG1 within the chitosan materials was confirmed by FTIR and XRD analyses. SEM analysis demonstrated that DOPA‐PonG1 had minimal effect on the surface morphology of the material, whereas the addition of rGO resulted in the formation of increased particle number on the chitosan surface, thus greatly enhancing its roughness. This increase in surface roughness provides more sites for cell attachment and, to some measure, improves the material's cytocompatibility. Furthermore, the material's hydrophilicity is critical to its biological activity, as a hydrophilic surface promotes cell attachment and motility.^16^ Both rGO and DOPA‐PonG1 increased the hydrophilicity of chitosan materials through a synergistic effect. This is because the hydrophilic properties of DOPA‐PonG1 and the peculiar structure of rGO promote water diffusion. Moreover, when rGO was added to the CS material, it significantly improved its tensile strength, while DOPA‐PonG1 had little effect on the mechanical properties of the material itself. Thus, these alterations improved the materials' hydrophilicity and mechanical strength, both of which are key considerations in wound healing applications.^17^

In addition to surface structure, hydrophilicity, and mechanical strength, the antibacterial property of wound dressing materials are critical to their efficiency.^18^ Antibacterial dressing materials can help limit the formation of harmful bacteria around the wound, producing a favorable environment for wound healing. We observed that the combination of rGO and DOPA‐PonG1 significantly increased the antibacterial efficacy of CS. Within 12 h, the combined material exhibited an antibacterial rate close to 100%. Taken together, the inclusion of rGO and DOPA‐PonG1 was effective in improving the CS material's overall physical and chemical properties, as well its antibacterial activity.

Cell growth and tissue regeneration occur in the human body alongside intricate microcurrent activities, and studies have shown that microcurrent electrical stimulation favors wound healing.^19^, ^20^ Electrical microcurrent treatment uses low intensity (μA) currents that are comparable to endogenous electric fields produced during wound healing, and supports the formation of extracellular matrix proteins whose formation and diffusion promote cell adhesion and proliferation.^21^ Furthermore, research has shown that combining electroactive materials with electrical stimulation might have a synergistic effect, improving therapeutic efficacy in skin injury.^22^ We observed that DOPA‐PonG1@CS/rGO materials exhibited superior cell viability and adhesion capabilities compared to unmodified CS materials likely due to the increased expression of genes required for tissue repair including matrix protein (Col1) and angiogenesis (VEGF). We identified that application of 100 mV electrical stimulation was most effective in stimulating cell viability, and when electrical stimulation was combined with DOPA‐PonG1@CS/rGO materials, there was further acceleration in wound healing.

Finally, we confirmed the efficacy of the DOPA‐PonG1@CS/rGO material and electrical stimulation therapy in promoting skin tissue regeneration and accelerating wound healing in a rat model of skin injury. The addition of DOPA‐PonG1@CS/rGO material significantly improved the tissue repair ability of CS, as demonstrated by the enhanced healing rate of epidermal wounds compared to CS alone. Furthermore, the combination of DOPA‐PonG1@CS/rGO material and electrical stimulation accelerated healing even further, with wound closure approaching 100% by the 9^th^ day. Histological analysis showed that CS, CS/rGO, and DOPA‐PonG1@CS groups exhibited thicker but disordered newly formed epidermis, indicating incomplete repair. In contrast, the DOPA‐PonG1@CS/rGO and DOPA‐PonG1@CS/rGO+ES groups exhibited the formation of thinner, flat, and orderly arranged tissue, indicating a superior repair effect. The Masson staining data showed greater collagen deposition in the newly formed tissues of the DOPA‐PonG1@CS/rGO and DOPA‐PonG1@CS/rGO+ES groups. Since collagen is the main structural protein in the extracellular matrix and plays a key role in wound healing by providing mechanical strength and support to healing tissue, our finding that the combination of DOPA‐PonG1@CS/rGO material and electrical stimulation therapy enhance the regenerative capacity and overall effectiveness of wound healing.

In conclusion, we developed a novel electroactive antibacterial material, DOPA‐PonG1@CS/rGO by incorporating rGO and DOPA‐PonG1 into CS. The material demonstrated excellent hydrophilicity, mechanical properties, and antibacterial activity. Cell and animal studies demonstrated that when combined with electrical stimulation, this material significantly promoted cell viability, adhesion, expression of tissue repair‐related genes, and accelerated skin tissue regeneration. These findings have therapeutic implications for the use of electroactive antibacterial materials and electrostimulation therapy, providing a novel approach to skin defect repair.

## Data Availability

The data that support the findings of this study are available from the corresponding author upon reasonable request.
